# Larvicidal activity of neem oil (*Azadirachta indica*) formulation against mosquitoes

**DOI:** 10.1186/1475-2875-8-124

**Published:** 2009-06-08

**Authors:** Virendra K Dua, Akhilesh C Pandey, Kamaraju Raghavendra, Ashish Gupta, Trilochan Sharma, Aditya P Dash

**Affiliations:** 1 National Institute of Malaria Research, Field Unit, Sector-III, BHEL, Hardwar 249043, India; 2 National Institute of Malaria Research, 22 Sham Nath Marg, Delhi 110054, India

## Abstract

**Background:**

Mosquitoes transmit serious human diseases, causing millions of deaths every year. Use of synthetic insecticides to control vector mosquitoes has caused physiological resistance and adverse environmental effects in addition to high operational cost. Insecticides of botanical origin have been reported as useful for control of mosquitoes. *Azadirachta indica *(Meliaceae) and its derived products have shown a variety of insecticidal properties. The present paper discusses the larvicidal activity of neem-based biopesticide for the control of mosquitoes.

**Methods:**

Larvicidal efficacy of an emulsified concentrate of neem oil formulation (neem oil with polyoxyethylene ether, sorbitan dioleate and epichlorohydrin) developed by BMR & Company, Pune, India, was evaluated against late 3^rd ^and early 4^th ^instar larvae of different genera of mosquitoes. The larvae were exposed to different concentrations (0.5–5.0 ppm) of the formulation along with untreated control. Larvicidal activity of the formulation was also evaluated in field against *Anopheles*, *Culex*, and *Aedes *mosquitoes. The formulation was diluted with equal volumes of water and applied @ 140 mg *a.i*./m^2 ^to different mosquito breeding sites with the help of pre calibrated knapsack sprayer. Larval density was determined at pre and post application of the formulation using a standard dipper.

**Results:**

Median lethal concentration (LC_50_) of the formulation against *Anopheles stephensi*, *Culex quinquefasciatus *and *Aedes aegypti *was found to be 1.6, 1.8 and 1.7 ppm respectively. LC_50 _values of the formulation stored at 26°C, 40°C and 45°C for 48 hours against *Ae. aegypti *were 1.7, 1.7, 1.8 ppm while LC_90 _values were 3.7, 3.7 and 3.8 ppm respectively. Further no significant difference in LC_50 _and LC_90 _values of the formulation was observed against *Ae. aegypti *during 18 months storage period at room temperature. An application of the formulation at the rate of 140 mg *a.i*./m^2 ^in different breeding sites under natural field conditions provided 98.1% reduction of *Anopheles *larvae on day 1; thereafter 100% reduction was recorded up to week 1 and more than 80% reduction up to week 3, while percent reduction against *Culex *larvae was 95.5% on day 1, and thereafter 80% reduction was achieved up to week 3. The formulation also showed 95.1% and, 99.7% reduction of *Aedes *larvae on day 1 and day 2 respectively; thereafter 100% larval control was observed up to day 7.

**Conclusion:**

The neem oil formulation was found effective in controlling mosquito larvae in different breeding sites under natural field conditions. As neem trees are widely distributed in India, their formulations may prove to be an effective and eco-friendly larvicide, which could be used as an alternative for malaria control.

## Background

Mosquitoes transmit serious human diseases like malaria, filariasis, Japanese encephalitis, dengue haemorrhagic fever and yellow fever causing millions of deaths every year [1]. Extensive use of chemical insecticides for control of vector borne diseases has created problems related to physiological resistance to vectors, adverse environmental effects, high operational cost and community acceptance [2]. Numerous plant products have been reported either as insecticides for killing larvae or adult mosquitoes or as repellents for mosquito biting and are one of the best alternatives for mosquito control [2,3].

Neem trees, (*Azadirachta indica*) native of India, belonging to family Meliaceae are fast growing evergreen trees ranging in height from 12 – 24 m. They are widespread in tropical and subtropical regions of the world, including semi-arid and wet- tropical regions [4]. Neem seeds contain approximately 99 biologically active compounds of which azadirachtin, nimbin, nimbidin and nimbolides are major molecules. Many of these derived products have antifeedancy, ovicidal activity, fecundity suppression besides insect growth regulation and repellency against insects [5-10]. Neem products have low toxicity to birds, fish and mammals and are less likely to induce resistance due to their multiple mode of action on insects. In addition to this, insect growth regulatory activity of neem weakens the cuticle defence system of the larvae causing easy penetration of pathogenic organisms into insect system. Azadirachtin, a biologically active compound has been promoted as a new insecticide that is considered more eco- friendly than synthetic insecticides. The pesticidal efficacy, environmental safety and public acceptability of neem and its products for control of crop pests has led to its adoption into various mosquito control programmes [8,11].

The present study was aimed to determine the larvicidal potential of the emulsified neem oil formulation against different mosquito genera under natural field conditions in India.

## Methods

### Neem oil formulation

The test formulation was an emulsified concentrate containing 0.15% w/v azadirachtin, polyoxyethylene ether (emulsifier), sorbitan dioleate (surfactant) and epichlorohydrin (used as a stabiliser to protect the degradation of the formulation under exposure to sun light.), developed by BMR & Company, Pune, India was evaluated against late 3^rd ^and early 4^th ^instar larvae of different genera of mosquitoes.

### Larvicidal bioassay

Larvicidal bioassay of the formulation was performed on late 3^rd ^and early 4^th ^instar larvae of *Anopheles stephensi*, a primary vector of urban malaria,*Culex quinquefasciatus *a common vector of filariasis, and *Aedes aegypti *a common vector of dengue, dengue haemorrhagic fever and yellow fever. The larvae were obtained from laboratory-established colony as described earlier [12]. Twenty-five larvae were released into 500 ml glass beakers containing 250 ml distilled water. The larvae were provided a mixture of dog biscuit and yeast powder in a 3:2 ratio as nutrients and supplemented with different concentrations (0.5 to 5.0 ppm) of the formulation. The experiments were carried out at 26°C ± 2°C. Five replicates of each concentration were run under the same microclimatic conditions along with untreated control. Mortality of larvae was monitored at 24 hours. The percent corrected mortality was calculated using Abbott's formula [13] and Log probit analysis was used to determine the median lethal concentration (LC_50_)/90% lethal concentration (LC_90_) of the formulation.

### Stability test

Larvicidal bioassay of the neem oil-based formulation stored at 26°C, 40°C and 45°C for 48 hrs was evaluated against *Ae. aegypti *at different concentration (0.5–5.0 ppm) as per the method reported above. Five replicates of each concentration were run under the same microclimatic conditions along with untreated control. A total of 100 larvae of *Ae. aegypti *were exposed against each concentration. Further bioassay test of the neem oil formulation of different concentrations (0.5–5.0 ppm) stored at room temperature (26°C ± 2°C) was evaluated against *Ae. aegypti *larvae at three months interval period for 18 months. Three replicates of each concentration were carried out along with control.

### Field evaluation of larvicidal activity

Field evaluation of larvicidal activity of the neem oil formulation was carried out in two districts of Uttar Pradesh viz. Mathura and Kanpur and district Hardwar of the state of Uttarakhand. Initially a survey was carried out to ascertain the suitability of different breeding habitats of target species for the trial. Larvicidal efficacy was carried out against late 3^rd ^and early 4^th ^instar larvae of different genera of mosquitoes under natural field conditions.

Larval density was determined using standard dipper (300 ml capacity with 9 cm diameter) method. Treatment dose (140 mg *a.i*./m^2^) of the formulation was determined on the basis of the results of laboratory evaluation carried out against *Cx. quinquefasciatus *and *Ae. aegypti *[14]. Five liters of the neem oil formulation was mixed with equal volume of water to make a uniform suspension and applied to 53 m^2 ^surface area of breeding habitats through precalibrated knapsack sprayer.

Larval density/dip was recorded a day before application for both experimental and control habitats, thereafter observations were recorded at 24, 48 and 72 hr of post application. Further observations were made at weekly intervals for 3 weeks. Percent reduction was calculated for 3^rd ^& 4^th ^instar larvae and pupae using the formula.



C_1_, C_2 _are pre-treatment, post-treatment larval density in control whereas T_1_, T_2 _are pre-treatment post-treatment, immature density in experimental habitats respectively.

## Results

### Laboratory study

**Figure 1:**
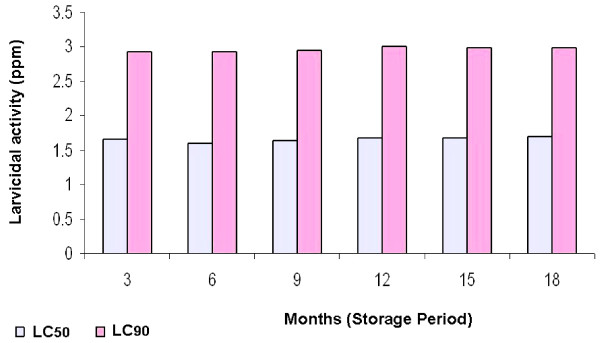
**Stability test of the neem oil formulation against *Aedes aegypti***.

Mean LC_50 _and LC_90 _values (95% confidence limits) of the neem oil formulation against *An. stephensi*, *Cx. quinquefasciatus *and *Ae, aegypti *are given in Table 1. Mean LC_50 _values of the formulation were 1.6, 1.8 and 1.7 ppm while LC_90 _were 3.4, 3.5 and 3.7 ppm against *An. stephensi*, *Cx. quinquefasciatus *and *Ae. aegypti *respectively. Results of stability test of the neem oil based formulation stored at different temperatures against *Ae. aegypti *are given in Table 2. LC_50 _values of the biopesticide stored at 26°C, 40°C and 45°C for 48 hours were 1.7, 1.7, 1.8 ppm while LC_90 _values recorded were 3.7, 3.7 and 3.8 ppm respectively. Mean LC_50 _and LC_90 _of the formulation during 18 months storage period at room temperature against *Aedes aegypti *is shown in Figure 1. No significant difference (P > 0.5) in LC_50 _and LC_90 _value of the formulation was observed.

**Table 1:** Larvicidal activity of neem oil formulation against mosquito in laboratory

**Species**	**Larvicidal activity (ppm)**	
	
	**LC**_50_ (Mean ± sd)	**LC**_90_ (Mean ± sd)
*Anopheles stephensi*	1.6 ± 0.4 (1.1 – 2.5)*	3.4 ± 0.5 (2.7 – 4.0)

*Culex quinquefasciatus*	1.8 ± 0.5 (1.2 – 2.6)	3.5 ± 0.6 (2.8 – 4.2)

*Aedes aegypti*	1.7 ± 0.3 (1.3 – 2.1)	3.7 ± 0.5 (3.1 – 4.3)

Water depth: 2.5 cm; * 95% confidence limits; Number of replicates: 5

**Table 2:** Larvicidal activity of neem oil formulation against *Aedes aegypti *at different storage temperature

**Storage temperature**	**Larvicidal activity (mean)**	
	
	**LC**_50_	**LC**_90_
26 ± 2°C	1.7 ± 0.5	3.7 ± 0.8

40°C	1.7 ± 0.6	3.7 ± 0.5

45°C	1.8 ± 0.4	3.8 ± 0.6

Number of each replicate: 3

### Field study

Before start of the study, a preliminary survey of various breeding sites of mosquitoes was carried out. In all 73 breeding sites (factory scraps) were surveyed inside the Ordnance factory, Kanpur, out of which 67 sites were found positive (91.8%) for *Aedes *larvae. Larvae collected from these breeding sites were identified as *Ae. aegypti *and *Ae. albopictus*. A survey of different breeding sites such as tanks, pits, drains were also carried at Indian Oil Corporation, Mathura and Bharat Heavy Electricals Limited, Hardwar for *Culex *and *Anopheles *breeding. *Culex *and *Anopheles *larvae collected from these sites were identified as *Cx. quinquefasciatus, An. culicifacies *and *An. subpictus *respectively.

**Figure 2:**
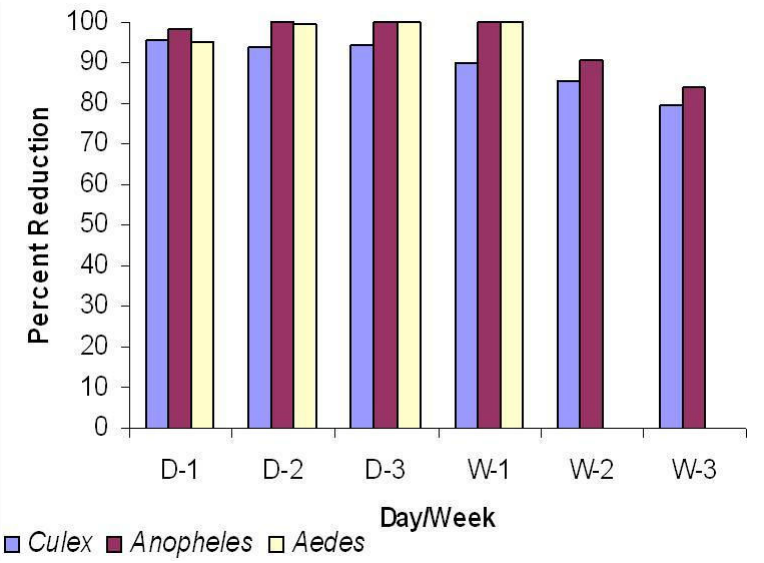
**Impact of larvicidal activity of the neem oil formulation against mosquito larvae**.

Mean percent reduction of larval density against *Cx. quinquefasciatus *and anophelines in different breeding habitats are given in Table 3. In pits, percent reduction of *Culex *larvae was 95.9, 90.2, 87.2 on days 1, 2, 3 respectively of post application, while more than 70% reduction was observed up to week 3. In tanks 91.6% – 92.4% reduction of *Culex *larvae was observed up to day 7 of post application thereafter 80.7% reduction was noted up to week 3, while in drains there was more than 90% larval control up to day 7 and remained above 75% up to week 3. An effective control of late instars anopheline larvae in tanks was observed with 98.2% reduction on day 1, followed by 100% reduction up to day 7 and more than 75% reduction till week 3. In pits, 96.2% control was recorded on day 1, 100% up to day 7 and more than 75% reduction up to week 3. The mean percent reduction of *Culex *larvae was 89.9–95.5% up to day 7 followed by 79.7–85.7% up to week 3, while for *Anopheles *larvae mean percent reduction was 90.4–100% up to day 7 followed by 83.8–90.4% up to week 3 (Figure 2).

**Table 3:** Larvicidal activity of neem oil formulation against mosquitoes larvae in field

***Mosquito species***	**Breeding sites**	**Pre treatment density**	**Percent reduction of larval density (mean ± sd)**					
			
			**Day-1**	**Day-2**	**Day-3**	**Week-1**	**Week-2**	**Week-3**
*Culex*	Pits	28.9 ± 10.6	95.9 ± 3.5	90.2 ± 6.9	87.2 ± 11.0	87.5 ± 8.2	85.9 ± 8.0	80.5 ± 7.3
	
	Tanks	26.8 ± 11.5	91.9 ± 5.8	93.2 ± 3.2	97.7 ± 1.9	92.4 ± 8.0	86.2 ± 8.2	80.7 ± 9.2
	
	Drains	115.7 ± 64.6	99.4 ± 0.6	98.8 ± 1.2	98.6 ± 1.4	84.9 ± 4.6	85.0 ± 11.8	77.8 ± 11.0

			**95.5 ± 4.1**	**94.1 ± 4.3**	**94.5 ± 5.5**	**89.9 ± 2.5**	**85.7 ± 0.8**	**79.7 ± 1.6**

*Anopheles*	Pits	13.5 ± 7.5	96.2 ± 4.5	100	100	100	85.4 ± 14.1	76.6 ± 9.6
	
	Tanks	10.4 ± 5.7	98.2 ± 1.8	100	100	100	87.0 ± 9.7	77.7 ± 10.0
	
	Drains	13.0 ± 6.7	100	100	100	100	98.7 ± 1.3	97.0 ± 3.0

			**98.1 ± 1.9**	**100**	**100**	**100**	**90.4 ± 7.2**	**83.8 ± 11.5**

Larvicidal activity of the formulation against *Aedes *larvae in different breeding sites is given in Table 4. There was 85.2% to 98.1% reduction of *Aedes *larvae on day 1 of post application of the neem oil formulation, thereafter 99.7% to 100% reduction was recorded up to day 7.

**Table 4:** Larvicidal activity of neem oil formulation against *Aedes *mosquitoes in field

Breeding sites	Pre treatment larval density	pH	Percent reduction of larval density (Mean ± sd)			
			
			Day-1	Day-2	Day-3	Day-7
Tyres	10.3 ± 4.1	8.0–9.0	94.3 ± 4.5	98.6 ± 1.4	100	100

Machinery scraps	14.5 ± 8.6	8.0–9.0	96.0 ± 3.0	100	100	100

Iron container	19.2 ± 5.7	8.5	98.1 ± 1.5	100	100	100

Iron box	11.0 ± 6.0	8.0–8.5	96.9 ± 2.0	100	100	100

Iron tanks	9.0 ± 2.6	8.0–8.5	85.2 ± 6.5	100	100	100

Plastic scrap	6.0	8.5	100	100	100	100

			**95.1 ± 5.2**	**99.7 ± 0.3**	**100**	**100**

Total replicates: 21

## Discussion

Neem trees are found throughout India with a myriad of uses in medicine, as well as pest control [4]. Neem-based pesticides are now extensively used in agriculture practices all over the world. It contains azadirachtin, which is a predominant insecticidal active ingredient, having antefeedent, ovipositional deterrence repellency, growth disruption, sterility and larvicidal action against insects [6]. There are various reports of control of mosquito breeding under field conditions. An emulsion of neem oil in water was found to be effective in controlling breeding of *Cx. quinquefasciatus*, *An. stephensi *and *Ae. aegypti *in pools, tanks and coolers up to 2 to 3 weeks [15], whereas an application of neem cake powder resulted in drastic reduction in the late instar larvae and pupae of culicine mosquitoes in paddy field [16].

Mean LC_50 _values were 1.6, 1.8 and 1.7 ppm against *An. stephensi*, *Cx. quinquefasciatus *and *Ae. aegypti*, while LC_90 _were 3.4, 3.5 and 3.7 ppm respectively. LC_50 _of the formulation stored at 26°C, 40°C and 45°C for 48 hours were 1.7, 1.7, 1.8 ppm while LC_90 _values were 3.7, 3.7 and 3.8 ppm respectively which revealed that there was no difference in the biopestcidal activity of the neem oil formulation at different storage temperatures. No significant difference of larvicidal activity of the formulation was also observed during 18 months storage period at room temperature.

In the present study an application of the formulation at the rate of 140 mg *a.i*./m^2 ^in pits, tanks and drains provided above 90% reduction of *Culex *larvae up to week 1 and thereafter 80% reduction up to week 3, whereas 100% reduction was observed in *Anopheles *larvae up to week 1, after that more than 80% reduction was recorded up to week 3.

Dhar *et al *[17] demonstrated the inhibitory effect of neem oil volatiles on gonotropic cycle in *An. stephensi *and *An. culicifacies*. A neem oil formulation containing 32% neem seed oil (an equivalent of 0.03% azadirachtin), an emulsifier (5%) and 63% iso propanol (solvent) was investigated for its larvicidal activities against *An. gambiae *[18]. It was toxic to mosquito larvae with LC_50 _value of 11 ppm and also reported to possess insect growth regulators. Gianotti and co workers [19] used powdered seeds of neem trees and applied twice a week to known breeding sites for *An. gambiae *at the rate of 10 gm/m^2 ^of pool surface area for effective larval control. Azadirachtin acts as anti-ecdysteroid and kills larvae by growth inhibition effect [20]. In the present investigation, neem oil formulation was found effective to control mosquito larvae in different breeding habitats under natural field conditions and more than 80% reduction of *Anopheles*, *Culex *and *Aedes *larvae was observed up to three weeks of post application.

Neem-based biopesticides and neem extracts have a wide range of effects against insect pests including repellence, feeding, toxicity, sterility and growth regulator activity and are relatively safe towards non- target biota with only minimal risk of direct adverse effects on aquatic biota from contamination of water bodies [21,22]. Allelochemicals such as azadirachtin, nimbin, nimbidin, nimbolides, nimolic acid, salannin, melianttriol, azadirachtol present in neem affect the biochemical and physiological processes of insect system and nullify the insect detoxification mechanism thereby not allowing the pest to develop resistance. As an emulsifiable concentrate, the neem oil formulation had greatly reduced sized particles and evenly mixed within the water column with a few suspended particles on the water surface. The spread of these fine particles probably increased the efficacy of formulation.

Control of mosquito larvae becomes a very pertinent issue in controlling the rapid replication of mosquitoes in management of vector- borne diseases. In the present study, neem oil formulation showed promising larvicidal activity against important vectors of malaria, filaria, dengue, dengue haemorrhagic fever, yellow fever and chikungunya. Development of resistance in temephos and *Bacillus thuringiensis *is a matter of concern for operational use as larvicides. Although the present formulation may be more costly than other larvicidal agents, such as temephos and *B. thuringiensis*, it has the advantage of being eco-friendly, effective and ability to prevent the development of pest resistance.

## Conclusion

The neem oil formulation was found effective in controlling mosquito larvae in different breeding sites under natural field conditions. Neem oil formulations are relative less toxic, eco-friendly and insects are unable to develop resistance and may be used as an alternative to other pesticides for control of vector- borne diseases.

## Competing interests

The authors declare that they have no competing interests.

## Authors' contributions

VD: designed the study protocols, directed the larvicidal research. ACP: designed and performed the larvicidal trials collected and analysed the data. KR: Editing the manuscript

AG: Performed larvicidal trials drafted the manuscript TS: Field trials of the larvicide. APD; Coordination and organizational help to conduct the study. All authors read and approved the final manuscript.

## References

[B1] Mittal PK, Subbarao SK (2003). Prospects of using herbal products in the control of mosquito vectors. ICMR Bull.

[B2] Brown AWA (1986). Insecticide resistance in mosquitoes; a pragmatic review. J Am Mosq Control Assoc.

[B3] Sukumar K, Perich MJ, Boobar LR (1991). Botanical derivative in mosquito control: A Review. J Am Mosq Control Assoc.

[B4] National Research Council (1992). Neem: a tree for solving global problems. Report of an adhoc panel of the Board on Science and Technology for International Development.

[B5] Isman MB (2006). Botanical insecticides, deterrent and repellents in modern agriculture and an increasingly regulated world. Ann Rev Entomic.

[B6] Schmutterer H (1990). Properties of natural pesticides from the neem tree, *Azadirachta indica*. Ann Rev Entomol.

[B7] Locantoni L, Guisti F, Cristofaro M, Pasqualini L, Esposito F, Lupetti P, Habluetzel A (2006). Effect of neem extract on blood feeding oviposition and oocyte ultra structure in *Anopheles stephensi *Liston (Diptera: Culicidae). Tissue Cell.

[B8] Su T, Mulla MS (1998). Antifeedancy of neem products containing Azadirachtin against *Culex tarsalis *and *Culex quinquefasciatus *(Diptera: Culicidae). J Vector Ecol.

[B9] Sharma VP, Dhiman RC (1993). Neem oil as a sand fly (Diptera: Psychodidae) repellent. J Am Mosq Control Assoc.

[B10] Schmutterer H (2002). The neem tree (*Azadirachta indica*) and other Meliceous plants. Source of Unique Natural Products for Integrated Pest Management, Medicine, Industry and other porposes.

[B11] Su T, Mulla MS (1998). Ovicidal activity of neem products (azadirachtin) against *Culex tarsalis *and *Culex quinquefasciatus *(Diptera; Culicidae). J Am Mosq Control Assoc.

[B12] World Health Organization (1981). Instruction for determining the susceptibility or resistance of mosquito larvae.

[B13] Abbott WS (1925). A method of computing the effectiveness of an insecticide. J Econ Entomol.

[B14] Chavassee DC, Yap NH, World Health Organization (1997). Chemical methods for the control of vector and pests of public health importance.

[B15] Batra CP, Mittal PK, Adak T, Sharma VP (1998). Efficacy of neem-water emulsion against mosquito immatures. Indian J Malariol.

[B16] Rao DR, Reuben R, Venugopal MS, Nagasampgi BA, Schmutterer H (1992). Evaluation of neem – *Azadirachta indica *with and without water management for the control of culicine mosquito larvae in rice field. Med Vet Entomol.

[B17] Dhar R, Dawar H, Garg SS, Basir F, Talwar GP (1996). Effect of volatiles from neem and other natural products on gonotropic cycle and oviposition of *Anopheles stephensi *and *An. culicifacies*. J Med Entomol.

[B18] Okumu FO, Knols BGJ, Fillinger U (2007). Larvicidal effects of a neem (*Azadirachta indica*) oil formulation on the malaria vector *Anopheles gambiae*. Malar J.

[B19] Gianotti RL, Bomblies A, Dafalla M, Issa-Arzika I, Duchemin JB, Eltahir EAB (2008). Efficacy of local neem extracts for sustainable malaria vector control in an African village. Malar J.

[B20] Zebit CPW (1984). Effect of some crude and *Azadirachta*-enriched neem (*Azadirachta indica*) seed kernel extracts of larvae of *Aedes aegypti*. Entomol Exp Appl.

[B21] Kreutzweiser DP (1997). Non-target effects of neem based insecticides on aquatic invertebrates. Ecotoxicol Env Safety.

[B22] Goektepe I, Portier R, Ahmedna M (2004). Ecological risk assessment of neem based pesticides. J Env Sci Hlth B.

